# Fracture de Galeazzi chez l’enfant: à propos de 5 cas et revue de la littérature

**DOI:** 10.11604/pamj.2018.30.274.6290

**Published:** 2018-08-14

**Authors:** Yassine Nhamoucha, Mohammed Tazi, Othmane Alaoui, Hicham Abdellaoui, Karima Atarraf, Lamyae Chater, Mounir Arroud, Abderrahmane Afifi

**Affiliations:** 1 Service de Traumato-Orthopédie Pédiatrique, Hôpital Mère et Enfant, CHU de Fès, Maroc; 2 Service de Chirurgie Pédiatrique, CHU Hassan II, Fès, Maroc

**Keywords:** Articulation radio-cubitale distale, fracture de Galeazzi, Maroc, Distal radioulnar articulation, Galeazzi fracture, Morocco

## Abstract

La fracture de Galeazzi associe une fracture de la diaphyse radiale avec une luxation radio-ulnaire distale, le plus souvent dorsale. Il s’agissait d’une étude rétrospective réalisée dans le service d’orthopédie traumatologie pédiatrique de l’hôpital mère enfant au CHU Hassan II de Fès (Maroc). L’étude portait sur cinq enfants de sexe masculin dont quatre cas ont été traités orthopédiquement avec une réduction chirurgicale dans le dernier cas. Le recul moyen est de 24 mois (8-30).

## Abstract (en)

Galeazzi fracture describes a fracture of the radial diaphysis in association with distal radioulnar dislocation (most often dorsal). We conducted a retrospective study in the Department of Pediatric Orthopedic Trauma at the Mother-Child Hospital CHU Hassan II in Fez (Morocco). The study involved five male children. Four children underwent orthopedic treatment while one child underwent surgical reduction. Mean follow-up was 24 months (8-30).

## Introduction

La fracture de Galeazzi est l’une des plus rares fractures de l’enfant et l’adolescent. Elle associe une fracture du radius à une luxation radio-ulnaire distale. Cette lésion peut être méconnue. Son traitement chez l’enfant est généralement orthopédique, la réduction chirurgicale est parfois nécessaire. Nous rapportons dans ce travail l’expérience de notre service à propos de cinq cas dont les résultats du traitement étaient satisfaisants.

## Patient et observation

Il s’agit de cinq enfants de sexe masculin, âgés respectivement de 8, 9, 10, 13 et 15 ans (âge moyen de 11 ans). La fracture de Galeazzi a été identifiée chez 96 enfants admis pour des fractures déplacées de l’avant bras ou du radius (5%) sur une période de 5 ans. Le mécanisme lésionnel exact a été précisé chez quatre de nos patients, ou ils étaient victimes d’une chute avec réception sur la paume de la main, poignet en hyperextension. L’atteinte du membre droit était présente chez 3 patients.

**Figure 1:**
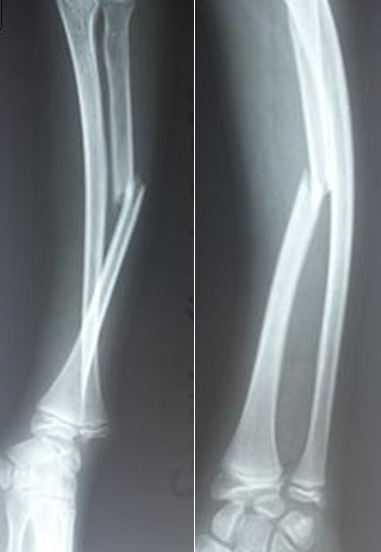
Images radiologique de l’avant bras face et profil objectivant une fracture du tiers moyen du radius avec luxation radio-ulnaire

Tous les malades présentaient une attitude du traumatisé du membre supérieur, avec une déformation de l’avant bras sans atteinte vasculo-nerveuse. Le bilan radiologique a permis le diagnostic mettant en évidence une fracture radiale medio-diaphysaire chez 3 enfants et du tiers distal de l’avant-bras chez les 02 autres, associées à une luxation radio-cubitale distale (Figure 1).

**Figure 2:**
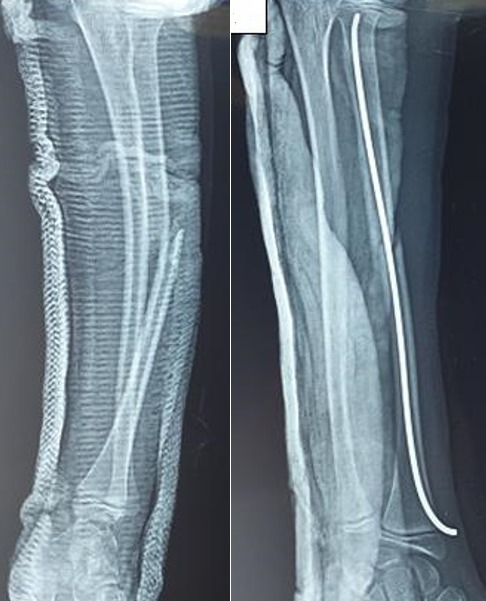
Images radiologique pré et post opératoire d’une fracture de galeazzi montrant la réduction chirurgicale avec embrochage centromédullaire élastique stable du radius et réduction de la luxation radio-ulnaire distale

La fracture radiale siégeait le plus souvent au tiers distal ou à sa jonction avec le tiers moyen. Les 04 premiers enfants ont été traités par une réduction orthopédique avec immobilisation par un plâtre brachio-antébrachio-palmaire pendant 06 semaines, le résultat était satisfaisant, avec un recul 26 et 14 mois. Le cinquième patient a bénéficié d’une réduction chirurgicale avec embrochage centromédullaire élastique stable du radius et réduction de la luxation radio-ulnaire distale (Figure 2), la radiographie de contrôle étant satisfaisante avec un recul de 50 jours.

## Discussion

Le diagnostic de la fracture de Galeazzi n’est pas difficile mais souvent méconnu. Très souvent la fracture du radius est notée, en revanche la luxation RUD est méconnue [1-3]. Décrite par Galeazzi en 1934 sur 18 cas, elle était déjà connue d’Astley Cooper en 1812 et a été décrite par Darrach en 1912 et Milch en 1926 [4]. Le mécanisme initial communément admis est un choc direct dorsoradial sur un avant-bras en pronation forcée, poignet en extension.

La variété postérieure de la luxation RCD prédomine dans toutes les séries [1, 5, 6]. En effet, au cours de l’hyperpronation, le cubital postérieur se luxe latéralement du fait de la rupture des points d’amarrage de sa gaine au radius, entraînant ainsi un glissement de la tête cubitale en arrière [1, 6].

Le traitement des fracture de galeazzi peut être divisé en fonction de l´âge. La gestion conservatrice a été trouvé de succès chez les enfants, bien que ce type de blessure est rare chez les enfants [3]. De bons résultats peuvent être obtenus chez les enfants lorsque le cubitus est déplacé dorsale (le plus commun) avec traction longitudinale, manipulation de la fracture, et immobilisation plâtrée en supination (4-6 semaines). En effet, dans cette position, la main se met en inclinaison radiale assurant une meilleure stabilité, et le carré pronateur joue un rôle actif de coaptation de la RCD [5].

Le déplacement ulnaire palmaire ou antérieure est moins fréquente [3, 5, 7]. La stabilité de ce type de fracture, après réduction, chez les enfants est présumé être dû à l´épaisseur du périoste. Aussi longtemps que la fracture est immobilisé dans un plâtre en supination un excellent résultat en découle. La réduction a ciel ouvert est parfois nécessaire si il ya des difficultés à maintenir la réduction; Toutefois, la fixation interne est généralement pas nécessaire [8-11].

## Conclusion

La fracture de Galeazzi est une entité rare, elle doit être suspectée devant toute fracture du radius. Son traitement chez l’enfant est souvent orthopédique, néanmoins la chirurgie peut être nécessaire.

## Conflits d’intérêts

Les auteurs ne déclarent aucun conflit d'interêts.
